# Design of Hardware Accelerators for Optimized and Quantized Neural Networks to Detect Atrial Fibrillation in Patch ECG Device with RISC-V

**DOI:** 10.3390/s23052703

**Published:** 2023-03-01

**Authors:** Ingo Hoyer, Alexander Utz, André Lüdecke, Holger Kappert, Maurice Rohr, Christoph Hoog Antink, Karsten Seidl

**Affiliations:** 1 Fraunhofer Institute for Microelectronic Circuits and Systems, 47057 Duisburg, Germany; 2 KIS*MED (AI Systems in Medicine), Technical University of Darmstadt, 64289 Darmstadt, Germany; 3 Department of Electronic Components and Circuits, University of Duisburg-Essen, 47057 Duisburg, Germany

**Keywords:** atrial fibrillation, artificial intelligence, quantization, neural networks, RISC-V

## Abstract

Atrial Fibrillation (AF) is one of the most common heart arrhythmias. It is known to cause up to 15% of all strokes. In current times, modern detection systems for arrhythmias, such as single-use patch electrocardiogram (ECG) devices, have to be energy efficient, small, and affordable. In this work, specialized hardware accelerators were developed. First, an artificial neural network (NN) for the detection of AF was optimized. Special attention was paid to the minimum requirements for the inference on a RISC-V-based microcontroller. Hence, a 32-bit floating-point-based NN was analyzed. To reduce the silicon area needed, the NN was quantized to an 8-bit fixed-point datatype (Q7). Based on this datatype, specialized accelerators were developed. Those accelerators included single-instruction multiple-data (SIMD) hardware as well as accelerators for activation functions such as sigmoid and hyperbolic tangents. To accelerate activation functions that require the e-function as part of their computation (e.g., softmax), an e-function accelerator was implemented in the hardware. To compensate for the losses of quantization, the network was expanded and optimized for run-time and memory requirements. The resulting NN has a 7.5% lower run-time in clock cycles (cc) without the accelerators and 2.2 percentage points (pp) lower accuracy compared to a floating-point-based net, while requiring 65% less memory. With the specialized accelerators, the inference run-time was lowered by 87.2% while the F1-Score decreased by 6.1 pp. Implementing the Q7 accelerators instead of the floating-point unit (FPU), the silicon area needed for the microcontroller in 180 nm-technology is below 1 mm${}^{2}$.

## 1. Introduction

Atrial Fibrillation (AF) is prevalent in about 2% of adults and one of the most common arrhythmias [1]. In Germany, AF is one of the top five reasons for hospitalization [2].

As McIntyre et al. found in their study with 2470 participants, 3% of patients above the age of 65 suffer from unknown AF. The condition is estimated to result in a stroke risk of 2% per year [3].

The probability of undetected AF increases with age, as Go et al. found in their study, including 17,974 adults. The prevalence among individuals over the age of 90 is estimated to be 9%, whereas it is only 0.1% among individuals below the age of 55. Over all strokes, AF is closely associated with causing about 15% [4].

AF in patients can indicate an increased risk of death and hospitalization with the next year, as Zink et al. point out in their study with 7107 patients [5].

To detect AF, Holter monitors are widely used. These devices are able to record an electrocardiogram (ECG) over a couple of hours for up to a few days. The recording time is crucial for identifying patients with AF.

Turakhia et al. investigated the benefits of an extended monitoring period for the detection of AF in a study with 26,751 patients. The study concluded that a recording time of 24 h can only identify 55.2% as many subjects compared to a recording over two weeks.

The assumption that a longer recording time is beneficial can be confirmed by Dagres et al.: investigating a group of 215 patients after catheter ablation, the study found that a 24 h Holter ECG only detected 59% of patients with AF compared to a 7-day recording.

As an alternative to today’s Holter monitors, some wearable devices, e.g., smart watches, are able to detect arrhythmias using integrated photoplethysmogram sensors [6]. Some wearables are also able to record ECG signals when triggered manually by the user [7]. Due to the high cost or missing medical certification, these devices are not suitable for publicly financed clinical applications.

As a compromise, modern patch ECG devices can be used. These devices are a lot smaller and more comfortable compared to Holter monitors. The patch ECG device is an ECG integrated in a band-aid with minimal weight. This kind of device is much more comfortable for patients who are supposed to wear the device for up to a few weeks. These devices are designed for single-use, reducing the effort in application for clinical staff. Additionally, modern patch ECG devices are able to collect ECG recordings of up to two weeks, though recording less channels than Holter monitors [8]. A one-channel ECG might still be sufficient to detect AF [7].

Compactness and longevity brings several challenges to the development of ECG patch devices. One of the main challenges is the demand for a low-cost solution, since the device is supposed to be used once and then disposed of. The enhanced recording period of up to several weeks with a light-weight power supply such as a lithium button cell battery requires a low-power hardware platform including microcontroller and memory, as well as specialized and optimized software.

Commercially available devices are usually supported by a PC or server for the analysis of the collected ECG recordings, since screening the data of several days is time intensive for physicians. For the analysis, artificial intelligence (AI) algorithms can be used to detect arrhythmias. The field of AI algorithms is broad. This work is focused on a fully connected NN as the structure offers opportunity for hardware acceleration.

NNs consist of individual neurons, inspired by the biological brain, that compute an output value depending on weighted inputs as well as an activation function. The weights of the NN have to be trained using labeled data. An NN at leas has an input and an output layer. Additional layers in-between are so-called hidden layers.

For the detection of arrhythmias in an ECG, even small neural networks with only one hidden layer have proven successful [9].

As inputs to the neural network, so-called features are computed first. These features can be in the time or frequency domain, as well as statistical variations. Commonly used are, e.g., the standard deviation of RR intervals or the median RR interval [10].

To reduce the energy consumption and allow real-time analysis of the data, integrating AI within the patch ECG device is beneficial. Due to the limited power supply, customized digital hardware, as well as optimized software, performing the neural network (NN) inference are developed.

Besides the main application of screening patients for AF with minimal impact on their daily life, the system may also be suitable for various other applications such as patient monitoring in clinics or nursing homes. The developed low-power AI solution may itself be transferred to industrial applications, such as battery powered sensors, and other vital signals, such as EEG, as well.

## 2. Objective, Motivation and Previous Work

To reduce energy consumption, cost and dimensions of the device, the system will be integrated as a system-on-chip (SoC) in an application specific integrated circuit (ASIC). Since memory is a critical factor in the silicon area needed for the ASIC, the memory requirements also have to be minimized. Estimating the minimal requirements for the hardware requires an analysis of the used algorithm and network topology [11].

As the device will be applied as a medical device, the reliable detection of AF is necessary. Classification of AF on a PC or server with today’s commercial software can achieve 55 to 98% in sensitivity and 84 to 97% specificity [1]. Since the presented approach is optimized for an embedded system, the goal of this work is to achieve 90% sensitivity and 80% specificity.

To reduce the clock frequency and therefore the energy consumption, the run time of inference in clock cycles has to be reduced. Common methods to reduce the computing effort are the quantization of the network as well as the simplification and elimination of calculations that do not contribute to the NN accuracy.

The datatype used is also relevant for energy consumption and cost. A 32-bit floating point multiplication may use as much as up to 18 times more energy than an 8-bit integer multiplication. Additionally, the area needed for the computing unit may be up to a 27 times increase in silicon area, which is an indicator of production cost [12].

As part of a previous BMBF pilot innovation competition [13], Fraunhofer IMS carried out research on a low power signal processing system to analyze the ECG data in terms of AF. The result was a low power ASIC for ECG signal processing, using an optimized NN. The network was implemented as a convoluted network, using hardware with parallel implemented multiply-accumulate units. The system performed one inference within 146 ms at a clock frequency of 12.5 MHz [14].

Since the NN was implemented in hardware, changes to the network after the ASIC synthesis are not possible. Therefore, adapting the NN to another application or optimizing the network structure for higher accuracy is not easy to realize. A software based approach, on the other hand, would allow future changes in the network structure, as well as the use of activation functions and signal preprocessing, including the methods used for feature extraction.

Thus, a detection of AF using a microcontroller and a software framework for embedded devices is a promising approach. As a follow-up project, the ARTEMIS project was initiated to investigate a software-based approach on a signal processing ASIC including a RISC-V-based microcontroller and on-chip memory in contrast to the fully hardware-based approach from the previous competition. In a previous work, the minimum requirements for this hardware platform were assessed [11] and presented at IEEE International Symposium on Medical Measurements and Applications. With respect to the identified quantized datatype, specialized hardware accelerators have been implemented in hardware description language (verilog) [15], which was presented at the Joint Annual Conference of the Austrian, German and Swiss Societies for Biomedical Engineering. This paper presents an overview of the design decisions for the digital part as well as the results for the ASIC implementation.

## 3. Development and Optimization of the NN

The first step towards an efficient detection is the development of a signal preprocessing algorithm. It preprocesses the signals and also chooses the best ECG channels, since the analog front-end is able to switch between several electrodes. The algorithm then segments the best signals into time windows and extracts a total of 13 scalar features from the recorded data. These 13 features contain information about the detectable and distinct sections of an ECG signal, such as QRS complex, P and T wave, as well as the related distances between the sections. The mean value, drift and many more criteria are also taken into account, including a data point for signal quality.

These features were then used to train a floating-point based NN (Section 3.1) as proof-of-concept. The net was then quantized (Section 3.2), expanded (Section 3.3) and simplified (Section 3.4).

The goal of the quantization and optimization process was to access minimal requirements in energy consumption, runtime and memory as well as to propose a suitable datatype for customized accelerators.

### Database

The data were acquired by Charité—Universitätsmedizin Berlin in the clinical trial “Telemedical Interventional Monitoring in Heart Failure” (TIM-HF, NCT00543881) and include 22,400 ECG recordings of 354 heart failure patients [16]. Charité is a partner of Fraunhofer IMS in the ARTEMIS project. For training, 16,000 samples were used. The remaining 6400 samples, 3200 positive and 3200 negative samples were used for testing.

### 3.1. Floating-Point-Based MicroNet

The 13 extracted features were used to train a so-called MicroNet, with only one hidden layer and two output nodes (Table 1). Since the labels for the data points were only two values, for AF or no AF, in the recording the two output nodes were normalized to the value range of 0 to 1, to match the labels using a softmax activation function. The classification then was assessed by which of the outputs were higher. The number of nodes in the hidden layer, as well as the activation function, have an impact on the accuracy of the NN. Empirically, 27 nodes in the hidden layer with a softsign activation function were sufficient to achieve the required accuracy levels (Table 2).

For inference and feature extraction, the AIRISC was used. The AIRISC was developed at Fraunhofer IMS and implements the RC32IMFC set of instructions [17]. Additionally, customized hardware can also be integrated into the AIRISC to further accelerate NN inference. It will be produced as an SoC later in the project, with special attention to memory and timing requirements. On the software side, the framework artificial intelligence for embedded systems (AIfES) was used [18]. For rapid prototyping purposes and the investigation of timing results, a Field-Programmable Gate Array (FPGA) board was used to estimate the performance of an ASIC implementation during use. Because of the lower frequency and higher energy consumption, an FPGA itself is not a suitable technology for the patch integrated solution.

The run-time increases when using a microcontroller without a floating-point unit (FPU, Table 2). The FPU is one of the largest modules in the silicon area needed or FPGA resources used. Hence, for the ASIC implementation a software approach that does not require an FPU would simplify the design and reduce the area footprint, power consumption and production cost. For a 32-bit floating point multiplication unit, 27 times more silicon area is needed compared to the 8-bit integer [12].

### 3.2. Direct Quantization Using AIfES

Besides using floating point datatypes, AIfES enables the use of functions for quantization as well as optimized functions for the inference of quantized NNs. It is possible to use it with 32-bit fixed-point quantization (Q31) or an 8-bit fixed-point quantization (Q7, Table 3). Since the memory required by the NN is costly to implement in the ASIC, the smaller 8-bit quantization was chosen to be used in this preliminary work. The framework quantizes all weights and bias coefficients, as well as the input automatically.

The network structure was kept identical and no retraining has been performed on the previous 32-bit floating-point (FP32) NN. Since even a quantization from FP32 to 8-bit floating-point (FP8) causes losses [19], it was to be expected that losses occur with the 8-bit fixed point integer quantization. The advantages are memory and timing requirements (Table 2).

### 3.3. Compensating Quantization Losses

One common way to compensate for quantization losses is to add more nodes or layers to the NN topology [20]. However, to implement an expanded MicroNet, more coefficients are necessary; thus, the network must be retrained. At the time of this study, AIfES was not able to perform the training itself. Therefore, the Python based Keras framework was used to train the expanded MicroNet [21]. Since the mathematical approximation for sigmoid is the most accurate in AIfES compared to the other activation functions, sigmoid was used in the hidden layer.

**Figure 1:**
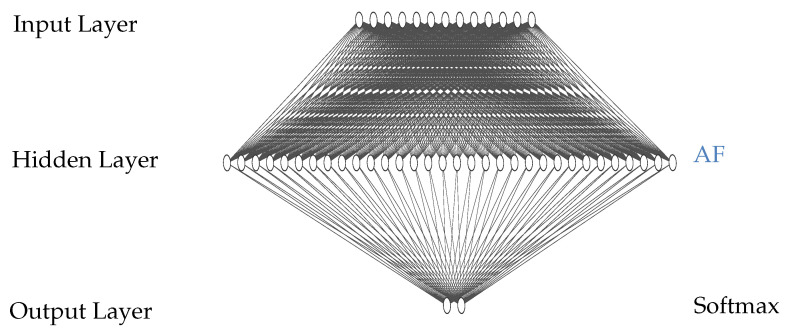
Schematic of the MicroNets used in this project to evaluate the hardware. Structure of the NNs is 13–32–2. The NNs differ in their activation function (AF) in the hidden layer, therefore marked in blue.

Empirically, the addition of five more nodes in the hidden layer results in acceptable accuracy (Table 2) in terms of the requirement after training in Keras. The new structure of the net is 13–32–2 (Figure 1), which later was also used to evaluate the specialized hardware. The training cycle was repeated until the sensitivity and specificity reached the required minimums.

After completion of training with Keras, the resulting coefficients are able to be directly used in AIfES. AIfES is optimized for embedded systems and has different approximations for mathematical functions, such as the sigmoid function. Due to this fact, the results of the MicroNet in AIfES are slightly different from the results in Keras. The expansion of the hidden layer results in slightly increased inference time and memory requirements (Table 2).

### 3.4. Simplification

The result of the output layer is directly and monotonically dependent on the input of the output nodes. Therefore, the result of the classification can already be retrieved from the sum of input values through the two output nodes. The activation function softmax is only necessary within training and fulfills the function of normalizing the incoming values to a range between 0 and 1. This is the reason why, in theory, the activation function of the output layer has no impact on the results of the network during inference. Thus, to further decrease memory and timing requirements, the network was simplified by changing the output activation function to rectifier linear unit (ReLU) as one of the fastest in the framework.

To further simplify the network, the output layer was reduced to one node. Without new training, the existing weights between hidden layer and output layer had to be subtracted, so that the resulting single output node can identify AF by the algebraic sign of the output value.

Since the NN has 13 input nodes and 32 nodes in the hidden layer, the first 13·32=416 weights, as well as the following 32 biases of the hidden layer, remain the same. Only the 64 weights for the connections between hidden and output layer have to be combined to 32 resulting weights $w_{r}$, by subtraction of the original weights $w_{o}$ (Equation (1)). The bias of the output layer itself also has to be combined. This recalculation can be described by the following formula:

(1) $$w_{r,j}=\left\{\begin{array}{cc} w_{o,i}, & i\leq 416\\ w_{o,i}-w_{o,i+1}, & i=2\;\cdot \;k>416,k\in N. \end{array}\right.$$

After the adjustments, the net now requires 449 weights $w_{r,j}$, where j∈[1,449]. To adjust the quota of sensitivity and specificity with regard to the criteria specified, the bias of the output nodes was manually adjusted from 0.53 to 0.9, which had a minimal impact on accuracy and F1-score (Table 2).

## 4. Specialized Hardware Accelerators

The basic structure of a generic NN consists of neurons and weighted connections between those (nodes and lines in the schematic representation in Figure 1). The input nodes are the features, the input values were extracted from the signal. The output nodes are the output values of the NN. In between these two groups of nodes (layers), hidden layers were used to process the input signals. Hidden layers and output layer in AIfES use activation functions to compute their output. Every line resembles a multiplication of the value of the previous node with a weight. The weighted inputs were then accumulated in the following nodes.

This general structure of neural networks allows many approaches to accelerate the use of the network for the classification of the input, the so-called inference. In this section, accelerators for neural network inference are presented that accelerate the activation functions in the nodes (Section 4.2) as well as the multiplications in-between (Section 4.3). The accelerators’ impact was then evaluated using small NNs.

The software framework AIfES features various activation functions. Commonly used are, e.g.,

- Sigmoid;
- Hyperbolic Tangent (tanh);
- Softmax; and
- Rectifier Linear Unit (ReLU).

ReLU only evaluates the sign of the input. If the input is positive, it is forwarded; otherwise, 0 is the output of the node. This principle is fast with the standard set of instructions; therefore, in this approach, it is not accelerated with specialized hardware.

Softmax function is defined as

(2) $$\sigma {\left(z\right)}_{i}=\frac{e^{z_{i}}}{\sum_{j=1}^{K}e^{z_{j}}}.$$

Due to the division, a complete hardware implementation of the softmax function would require a lot of resources. To accelerate the nodes using the softmax function, the e-function in the computation is accelerated using specialized hardware.

Sigmoid- and tanh-functions will be implemented in a combined hardware accelerator module as there is a mathematical relation between the two.

### 4.1. MicroNets for Evaluation

Since three different accelerators for activation functions were implemented, three similar NNs were trained to evaluate the performance of the hardware. Each of the networks have the same structure, using 13 input features and one hidden layer with 32 nodes as well as two output nodes (Figure 1). To reduce memory requirements, the NN was quantized to an 8-bit fixed-point datatype (Q7) in the software and corresponding hardware modules.

### 4.2. Accelerating the Nodes

In this section, the implementation of the hardware accelerator for the tanh-function is discussed. The goal of the chosen approach was to minimize hardware resources required. Hence, linearization, and as few data points in look-up tables as possible, were used.

Therefore a valid area for linearization,

(3) tanh(x)≈x,

is determined. The maximum error for the linearization was set to be below the quantization error of $\frac{1}{32}$, which is the lowest value represented in the Q7 interpretation of the hardware module (Table 3). Using MATLAB [22], the intersection of the according error band around the mathematical tanh-function with its linearization is found at x=0.4671.

The tanh-function has an asymptotic final value of

(4) $$\lim_{x\to \infty }\tan h\left(x\right)=1.$$

Therefore, to achieve maximum accuracy with the Q7 datatype, an approximation for the area between $x={\tan h}_{L}\left(x\right)=0.4671$ and tanh(x)=1 has to be found. Common methods for these approximations in hardware are look-up tables (LUTs), more specifically range addressable look-up tables (RALUTs) [23].

The difference between standard LUTs and RALUTs is that the values of an LUT are defined by its x-values. Especially with quantized datatypes, it is possible that several x-values correspond to the same y-value. This results in high memory requirements. Additionally, the error for the y-values is different for each x-value. In RALUTs, this problem is avoided by choosing the y-values first. The y-values are chosen equidistantly over the range that has to be covered. In this case, from 0.4671 to 1.

Due to the quantization, this results in 18 entries in the RALUT to cover every possible number.

**Figure 2:**
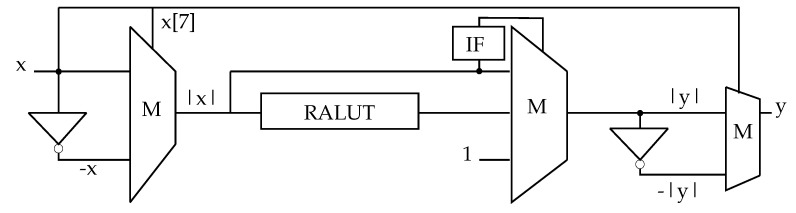
Schematic of the hardware that approximates the tanh-function. Due to the symmetry, the result is calculated for the absolute |x| of the input *x* choosing either a linearization, RALUT or border value.

**Figure 3:**
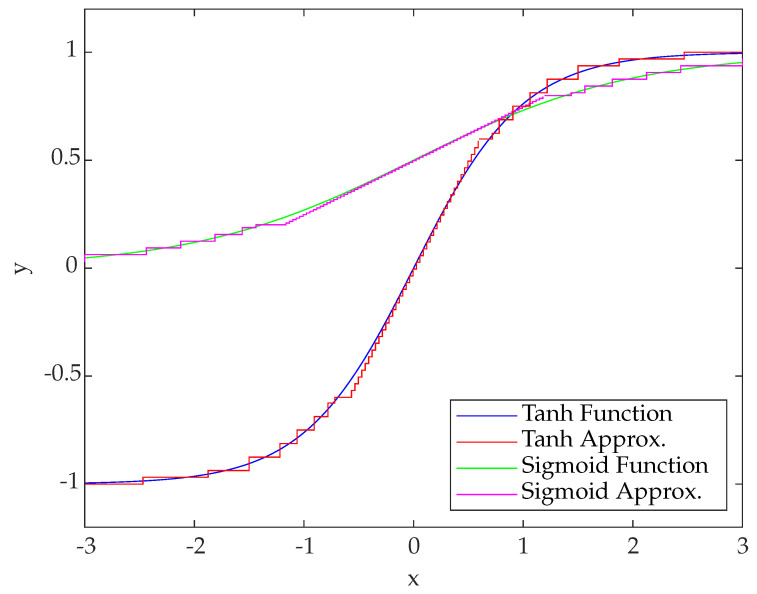
Plot of the mathematical hyperbolic tangent function (**blue**) and its hardware-implemented approximation (**red**), as well as the mathematical sigmoid function (**green**) with its approximation (magenta). Steps originate from the quantization (Q7) in the area of linearization, and from the RALUT.

Since the tanh-function is symmetric to the origin (Equation (5)), the same combination of linearization and RALUT can be used for negative input values. Therefore, multiplexers and inverters were used for the sign conversion (Figure 2). The results of the implementation are presented in Figure 3.

(5) tanh(x)=−tanh(−x).

The sigmoid function is approximated using the module for tanh and the following mathematical relation:

(6) $$S\left(x\right)=\frac{\tan h(\frac{x}{2})+1}{2}.$$

The division by two was implemented by shifting operations. The resulting approximation resembles the tanh-implementation because of the obvious combination of linearization and RALUT (Figure 3). The functionality of sigmoid and tanh-function were combined in one module, the submodule for the sigmoid function only implements the addition of one and the shifting operation of the output. The shifting operation at the input as well as multiplexing were performed in a combined module.

As mentioned, the softmax function was accelerated by accelerating the contained e-function in this approach. The accelerator for the e-function was restricted to the interval between between −1 and 1 for input values. If values outside this range occur in the program, they can still be calculated by multiplication with the Euler constant, respectively.

The approximation used is based on the cordic algorithm [24]. Therefore, it approximates the hyperbolic sine and cosine in several steps. Each computing step calculates

(7) $$x_{i+1}=x_{i}+\sigma_{i}y_{i}2^{-i},$$

(8) $$y_{i+1}=y_{i}+\sigma_{i}x_{i}2^{-i},$$

(9) $$z_{i+1}=z_{i}-\sigma_{i}\;\cdot \;\mathrm{atanh}\left(2^{-i}\right),$$

with

(10) $$\sigma_{i}=\left\{\begin{array}{cc} -1, & z_{i}<0\\ +1, & z_{i}>0. \end{array}\right.$$

The starting values are defined as

(11) $$x_{1}=P^{\prime },y_{1}=0,z_{1}=\phi .$$

$P^{\prime }=1.2075$ is a constant, ϕ is the input value [24]. For the inverse hyperbolic tangent (atanh), a small LUT is implemented for the relevant values.

The last y-value represents the sinh(ϕ), the last x-value the cosh(ϕ).

(12) $$y_{m+1}\approx \sin h\left(\phi \right),$$

(13) $$x_{m+1}\approx \cos h\left(\phi \right),$$

The result of the exponential function can be computed by Equation (14).

(14) $$\exp\left(\phi \right)=\left\{\begin{array}{cc} \cos h(\phi )-\sin h(\phi ), & \phi \leq 0\\ \cos h(\phi )+\sin h(\phi ), & \phi >0, \end{array}\right.$$

and therefore is approximated by Equation (15).

(15) $$\exp\left(\phi \right)\approx \left\{\begin{array}{cc} x_{m+1}-y_{m+1}, & \phi \leq 0\\ x_{m+1}+y_{m+1}, & \phi >0. \end{array}\right.$$

**Figure 4:**
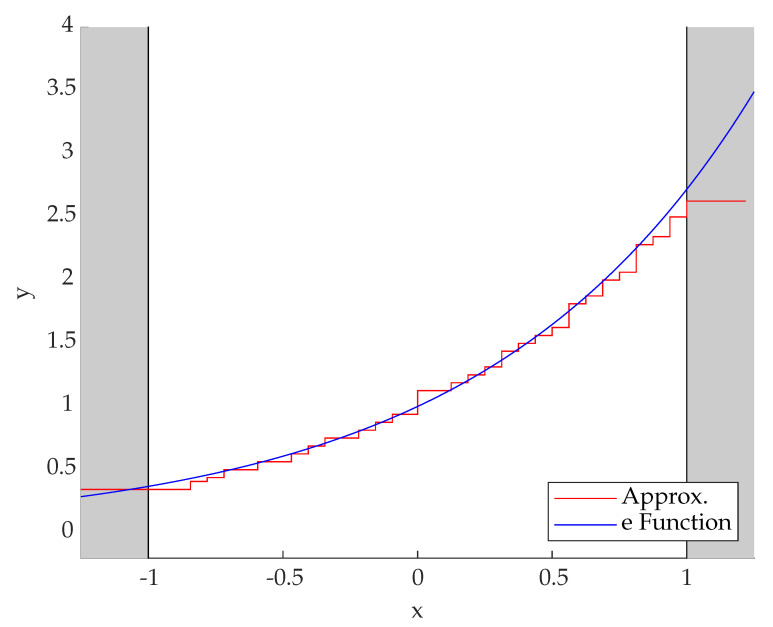
Plot of the mathematical e-function (**blue**) as well as the hardware-implemented approximation (**purple**). The algorithm is used in the range from −1 to 1.

In the presented hardware, three stages of the cordic algorithm were implemented. For a Q7 datatype, eight stages in total are necessary. The resulting approximation is shown in Figure 4. To achieve an operating speed of up to 100 MHz on the FPGA, the cordic algorithm was split into a three-step pipeline to reduce the timing critical paths.

### 4.3. Accelerating the Multiplications and Accumulations

The RISC-V P-Extension features various so-called single instruction multiple data (SIMD) operations. These operations can access 32-bit input registers, process multiple 8- or 16-bit values in parallel and write the result in a 64-bit result register. The result is then saved into memory in two 32-bit write operations.

For a Q7-based NN, up to four 8-bit multiplications in parallel may accelerate the inference by about a factor of 4. Hence, this is implemented as a modification of the existing multiplication module in the AIRISC.

**Figure 5:**
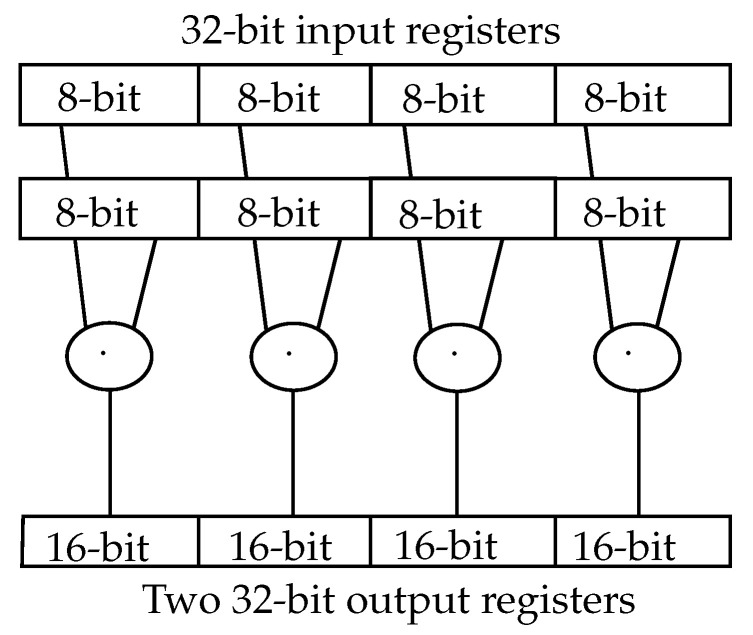
The SMUL8 instruction as an example of the SIMD instructions in the p-extension of the RISC-V ISA [25]. Eight 8-bit input values are accessed in two 32-bit words, multiplied and saved in four 16-Bit values in the 64-bit result.

To avoid having several redundant multiplication blocks, the multipliers can be combined to feature multiplications for bigger datatypes. The requirement for the 8-bit SIMD multiplication is four 8-bit multipliers (Figure 5). For future applications, a 16-bit SIMD is also integrated in the circuit using two 16-bit multipliers.

As the binomial equations (Equation (16)) illustrate, to correctly compute the result of a multiplication with numbers that are segmented in two, four multipliers and a three input adder for the results are needed.

(16) (a+b)·(c+d)=a·c+a·d+b·c+b·d.

**Figure 6:**
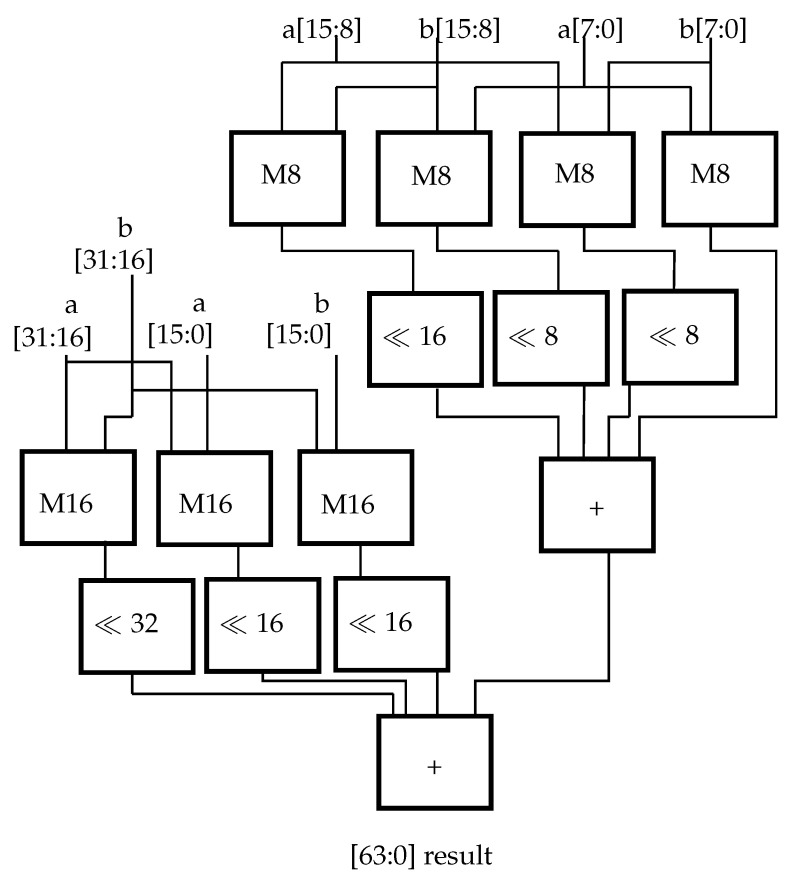
Combining the multipliers from 8- and 16-bit SIMD (M8, M16) together with an additional 16-bit multiplier to achieve a 32-bit multiplication in the 64-bit result. The result is then saved in two 32-bit write operations.

Hence, the four 8-bit multipliers can be connected to one 16-bit multiplier. Taking into account also the two 16-bit multipliers for 16-bit SIMD operations, three 16-bit operations can be executed in parallel by the hardware. To compute 32-bit multiplications with minimal additional hardware resources, one additional 16-bit multiplier is integrated as well as the adder for the result (Figure 6). To correctly sum the interim results, shifters have to be used as well.

## 5. Results and Discussion

**Figure 7:**
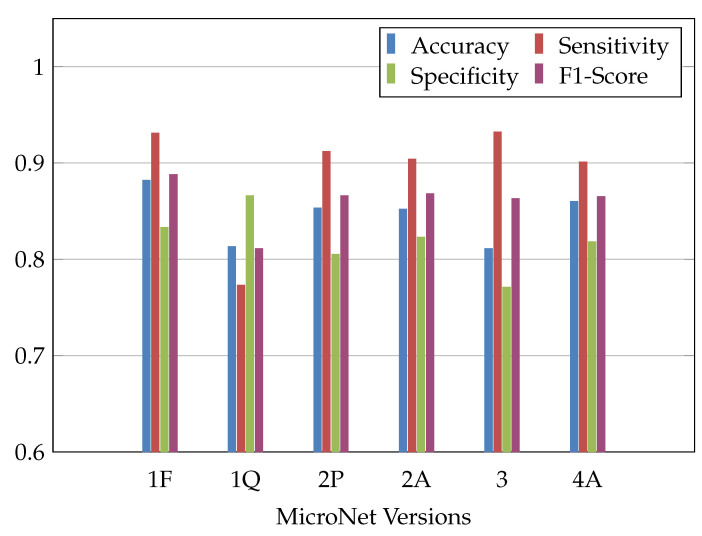
Accuracy (**blue**), sensitivity (**red**), specificity (**green**) and F1-Score (**purple**) for the different versions of the MicroNet.

Though the original, floating-point-based NN shows the highest F1-score with 88.8% (1F, Table 2), after quantization and optimization most of the loss is compensated, resulting in an increase of F1 score 2.3 percentage points (pp) less than the original (4A, Table 2, Figure 7). Percentage points are used to express a difference between two percentage values using subtraction. In runtime for the inference, 7.5% are saved. Memory requirements were reduced by 64.9%.

**Figure 8:**
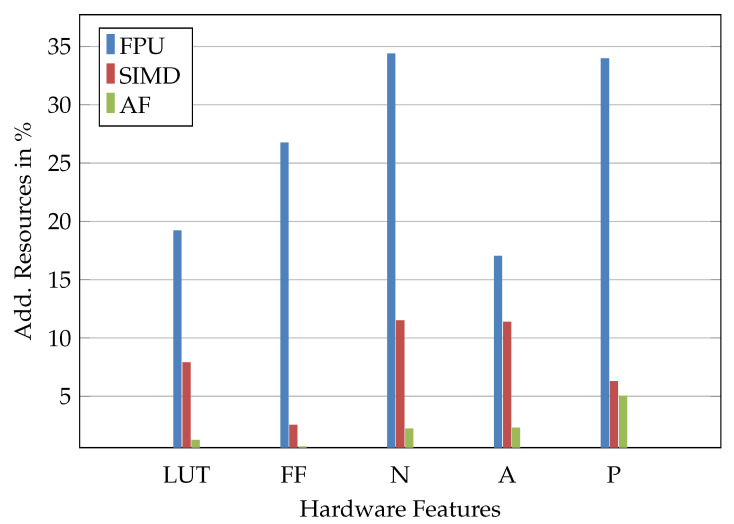
Additional look-up tables (LUT), flip-flops (FF), NAND equivalents (N), silicon area (A) and power (P) needed for the SIMD accelerators (SIMD), the accelerators for the activation functions (AF) and the floating point unit (FPU).

The designed hardware accelerators for a hyperbolic tangent and sigmoid show a maximum absolute error lower than two times the value of the least significant bit. The maximum error for the e-function is within 0.1912 for the range up to *e* satisfactory (Table 4). With regard to the hardware effort, the accelerators for SIMD and activation functions combined require less in all criteria (Figure 8, Table 5). The results for an ASIC synthesis are presented in Table 6. The accelerators achieved a reduction in runtime of up to 85.6% (SFS/SFH, Table 7) and as little as 9489 clock cycles (cc). The maximum F1-Score of the NN for testing was 86.83% (SGS, Table 7).

**Figure 9:**
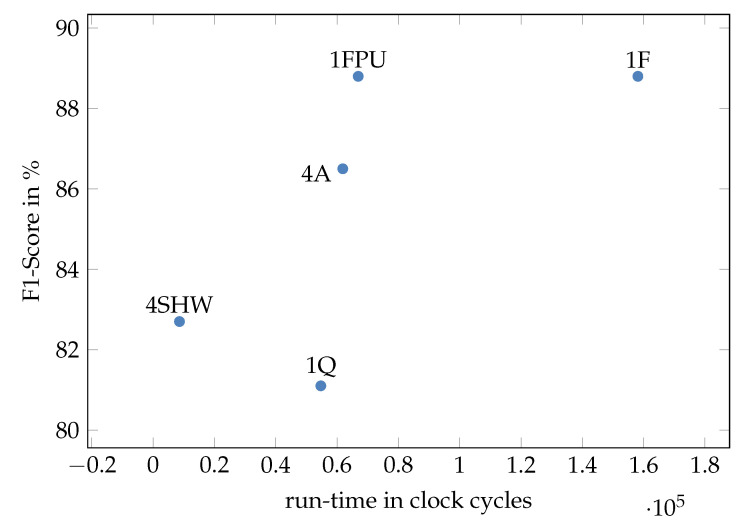
F1-Score (*y*-axis) vs. run-time in clock cycles on the AIRISC plotted for the different MicroNet versions. The FP-based MicroNet (1F) achieves the highest F1-Score, though also requires highest run-time. The extended, quantized net (4A) compensates most of the quantization loss in the directly quantized MicroNet (1Q). The specialized accelerate reduce runtime a lot (4SHW).

Using the specialized hardware on the previously optimized NN (4A, Figure 9) achieved the lowest runtime in inference with 8587 cc but at a loss of 6.1 pp (4SHW) compared to the floating-point-based micronet (1F/1FPU). This may come from the error of the hardware approximations.

**Figure 10:**
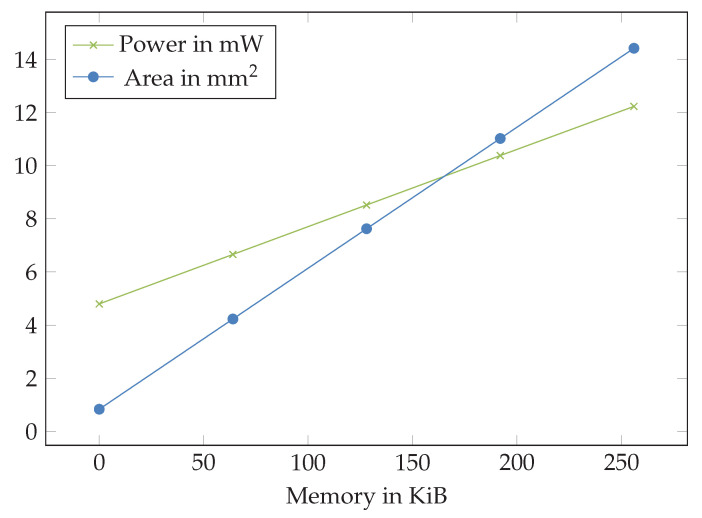
Impact of memory installed with an AIRISC microprocessor to power consumption and silicon area needed.

An on-chip memory of 64 kB appears to be realistic for the inference, as the combined inference results and model parameter memory is below 1 kB and the program memory can be reduced below 56.708 kB. The memory has a significant impact on silicon area and power consumption (Figure 10).

If the inference should be executed once per second, a clock frequency of 8.6 kHz would be sufficient with the specialized accelerators. However, this only covers the inference, not signal preprocessing or feature extraction. These additional calculations were responsible for up to 54% of energy consumption in the *Hinkelstein* project [14]. The impact in this work is expected to be similar.

## 6. Conclusions and Outlook

Due to the positive results presented with the described accelerator architecture, the ASIC may be implemented without an FPU. The performance of the NN may be further optimized by using the hardware approximations in training. An adaption or optimization of the feature extraction to the analog frontend used in the device might also prove to be beneficial. As the quality of ECG recordings was good, the device has to be tested in the field to prove the performance.

Besides the power consumption, memory required is assumed to be the limiting factor for AI algorithms on the SoC as it has a significant impact on silicon area.

If the evaluation of the developed device is positive, it can be adapted to several applications, e.g., for telemedical observation of patients, informing medical staff if necessary.

Future approaches to optimizing the AIRISC for AI algorithms could be supplementary accelerators for even smaller datatypes, as sub-byte-to-single-bit datatypes have proven useful for some applications [26]. An other approach could be the use of a small embedded FPGA to allow reconfiguration of the device with respect to the application, as it was implemented with different RISC-V cores [27].

## Figures and Tables

**Table 1:** Different MicroNet versions with their structures.

Net	Data	Run in	Structure	Activ. Funct.		Notice
Abbrv.	Type			HL	Out.	
1F	FP32	AIfES	13–27–2	Softsign	Softmax	w. sw float
1FPU	FP32	AIfES	13–27–2	Softsign	Softmax	w. FPU
1Q	Q7	AIfES	13–27–2	Softsign	Softmax	
2P	Q7	Python	13–32–2	Sigmoid	Softmax	
2A	Q7	AIfES	13–32–2	Sigmoid	Softmax	
3	Q7	AIfES	13–32–2	Sigmoid	ReLU	
4	Q7	AIfES	13–32–1	Sigmoid	ReLU	
4A	Q7	AIfES	13–32–1	Sigmoid	ReLU	Adj. Offset
4SHW	Q7	AIfES	13–32–1	Sigmoid	ReLU	Special HW

**Table 2:** Different MicroNet versions with their results in accuracy, sensitivity, specificity, F1-Score, parameter memory, flexible memory and run-time. Arrows indicating the goal for optimization.

Net	Acc.	Sens.	Spec.	F1	P. Mem.	Fl. Mem	Run-Time
Abbrv.	in %	in %	in %	in %	in Bytes	in Bytes	in cc
	↑	↑	↑	↑	↓	↓	↓
1F	**88.2**	93.1	83.3	**88.8**	1736	216	158,122
1FPU	88.2	93.1	83.3	**88.8**	1736	216	66,894
1Q	81.3	77.3	**86.6**	81.1	**560**	**64**	**54,695**
2P	85.3	91.2	80.5	86.6	N. A.	N. A.	N. A.
2A	85.2	90.4	82.3	86.8	652	72	66,295
3	81.1	**93.2**	77.1	86.3	652	72	65,943
4	86.0	86.5	85.6	86.1	**616**	**72**	61,857
4A	86.0	90.1	81.8	86.5	**616**	**72**	**61,857**
4SHW	81.6	87.8	75.4	82.7	**616**	**72**	**8587**

**Table 3:** Q7 datatype in the hardware modules.

	Sign	Integer		Fragments				
Bit-Number	7	6	5	4	3	2	1	0
Value	+/−	2	1	$\frac{1}{2}$	$\frac{1}{4}$	$\frac{1}{8}$	$\frac{1}{16}$	$\frac{1}{32}$

**Table 4:** Maximum absolute error in the x-range of x∈[−1:1] for the accelerators compared to the mathematical function.

Accelerator	Error
tanh(x)	0.0610
S(x)	0.0320
exp(x)	0.1912

**Table 5:** Resources needed for the additional hardware on the FPGA. The relative values are referring to the AIRISC without the FPU, accordingly 11,350 LUTs and 4557 FFs.

Functionalities	Add. LUTs	Add. FF	rel. LUTs	rel. FF
Tanh-, Sgm-, e-Fct.	171	41	1.5%	0.90%
SIMD	1416	161	12.4%	3.53%

**Table 6:** Synthesis results for the ASIC in 180 nm technology.

Width	975 μm
Length	975 μm
Area	$0.951\;{\mathrm{mm}}^{2}$
NAND-Eq.	61,150
Max. Frequency	30 MHz

**Table 7:** Results for the three MicroNets with their different activation function, abbreviation (Abrv.) for Figure 9, if hardware accelerators were used (HW) or purely software activation functions, runtime (RT) without and with SIMD in clock cycles as well as accuracy, Sensitivity, specificity and F1-Score to evaluate the performance of the hardware. Arrows indicating the direction of optimization.

Activation	Abrv.	HW or	RT w/o	RT w.	Acc.	Sens.	Spec.	F1
Function		SW	SIMD	SIMD				
		Used	in cc	in cc	in %	in %	in %	in %
			↓	↓	↑	↑	↑	↑
**Tanh**	THS	SW	63,463	10,106	82.63	78.29	**86.97**	81.84
	THH	HW	63,180	**9489**	81.69	78.16	85.21	81.02
**Sigmoid**	SGS	SW	66,312	12,077	85.23	90.34	82.25	**86.83**
	SGH	HW	**58,418**	10,073	79.31	**92.26**	69.86	82.97
**Softmax**	SFS	SW	67,538	11,166	84.17	86.02	84.78	85.49
	SFH	HW	65,998	9700	**86.03**	87.04	85.03	86.17

## Data Availability

The datasets provided by Charité—Universitätsmedizin Berlin are not publically available due to ethical and privacy restrictions.

## Abbreviations

The following abbreviations are used in this manuscript:

AF	Artrial Fibrillation
AI	Artificial Intelligence
ASIC	Application Specific Integrated Circuit
cc	Clock Cycles
FF	Flip-Flop
FPGA	Field Programmable Gate Array
FPU	Floating Point Unit
FP8	8-bit floating-point datatype
FP32	32-bit floating-point datatype
LUT	Look-Up Table
N	NAND Equivalents
NN	Neural Network
pp	Percentage Points
Q7	8-bit fixed-point datatype
Q31	32-bit fixed-point datatype
RALUT	Range Addressable Look-Up Table
ReLU	Rectifier Linear Unit
RT	Runtime
SIMD	Single-Instruction Multiple Data
SoC	System-On-Chip

## References

[1] Sajeev J.K., Koshy A.N., Teh A.W. (2019). Wearable devices for cardiac arrhythmia detection: A new contender?. Intern. Med. J..

[2] Augurzky B., Decker S., Leber R., Mensen A. (2021). Barmer Krankenhausreport 2021—Krankenhausinfektionen Während der COVID-19-Pandemie im Jahr 2020. https://www.barmer.de/resource/blob/1032118/6daf6b984a03df138b5bd69d98a685de/pressemappe-barmer-krankenhausreport-2021-krankenhauskeime-data.pdf.

[3] McIntyre W.F., Wang J., Benz A.P., Johnson L., Connolly S.J., Van Gelder I.C., Lopes R.D., Gold M.R., Hohnloser S.H., Lau C.P. (2022). Estimated incidence of previously undetected atrial fibrillation on a 14-day continuous electrocardiographic monitor and associated risk of stroke. EP Europace.

[4] Go A.S., Hylek E.M., Phillips K.A., Chang Y., Henault L.E., Selby J.V., Singer D.E. (2001). Prevalence of diagnosed atrial fibrillation in adults: National implications for rhythm management and stroke prevention: The AnTicoagulation and Risk Factors in Atrial Fibrillation (ATRIA) Study. JAMA.

[5] Zink M.D., Mischke K.G., Keszei A.P., Rummey C., Freedman B., Neumann G., Tolksdorf A., Frank F., Wienströer J., Kuth N. (2020). Screen-detected atrial fibrillation predicts mortality in elderly subjects. EP Europace.

[6] Turakhia M.P., Desai M., Hedlin H., Rajmane A., Talati N., Ferris T., Desai S., Nag D., Patel M., Kowey P. (2019). Rationale and design of a large-scale, app-based study to identify cardiac arrhythmias using a smartwatch: The Apple Heart Study. Am. Heart J..

[7] Bumgarner J.M., Lambert C.T., Hussein A.A., Cantillon D.J., Baranowski B., Wolski K., Lindsay B.D., Wazni O.M., Tarakji K.G. (2018). Smartwatch Algorithm for Automated Detection of Atrial Fibrillation. J. Am. Coll. Cardiol..

[8] Turakhia M.P., Hoang D.D., Zimetbaum P., Miller J.D., Froelicher V.F., Kumar U.N., Xu X., Yang F., Heidenreich P.A. (2013). Diagnostic utility of a novel leadless arrhythmia monitoring device. Am. J. Cardiol..

[9] Plesinger F., Nejedly P., Viscor I., Halamek J., Jurak P. Automatic detection of atrial fibrillation and other arrhythmias in holter ECG recordings using rhythm features and neural networks. Proceedings of the 2017 Computing in Cardiology (CinC).

[10] Rizwan A., Zoha A., Mabrouk I.B., Sabbour H.M., Al-Sumaiti A.S., Alomainy A., Imran M.A., Abbasi Q.H. (2020). A review on the state of the art in atrial fibrillation detection enabled by machine learning. IEEE Rev. Biomed. Eng..

[11] Hoyer I., Utz A., Lüdecke A., Richter M., Wichum F., Gembaczka P., Köhler K., Rohr M., Hoog Antink C., Seidl K. Detection of atrial fibrillation with an optimized neural network on a RISC-V-based microcontroller for efficient integration into ECG patches. Proceedings of the 2022 IEEE International Symposium on Medical Measurements and Applications (MeMeA).

[12] Gholami A., Kim S., Dong Z., Yao Z., Mahoney M.W., Keutzer K. (2021). A Survey of Quantization Methods for Efficient Neural Network Inference. arXiv.

[13] Hans-Joachim Rickel B. Pilotinnovationswettbewerb Energieeffizientes KI-System. https://www.elektronikforschung.de/service/aktuelles/pilotinnovationswettbewerb.

[14] Lerch R., Hosseini B., Gembaczka P., Fink G.A., Lüdecke A., Brack V., Ercan F., Utz A., Seidl K. Design of an Artificial Neural Network Circuit for detecting Atrial Fibrillation in ECG Signals. Proceedings of the 2021 IEEE Sensors.

[15] Hoyer I., Utz A., Lüdecke A., Rohr M., Hoog Antink C., Seidl K. (2022). Inference runtime of a neural network to detect atrial fibrillation on customized RISC-V-based hardware. Curr. Dir. Biomed. Eng..

[16] Koehler F., Winkler S., Schieber M., Sechtem U., Stangl K., Böhm M., Boll H., Baumann G., Honold M., Koehler K. (2011). Telemedical Interventional Monitoring in Heart Failure Investigators. Impact of remote telemedical management on mortality and hospitalizations in ambulatory patients with chronic heart failure: The telemedical interventional monitoring in heart failure study. Circulation.

[17] (2021). AIRISC System-On-Chip Design. https://www.airisc.de.

[18] (2022). Artificial Intelligence for Embedded Systems. https://www.aifes.de.

[19] Sun X., Choi J., Chen C.Y., Wang N., Venkataramani S., Cui X., Zhang W., Gopalakrishnan K. (2019). Hybrid 8-bit floating point (HFP8) training and inference for deep neural networks. Adv. Neural Inf. Process. Syst..

[20] Foody G.M., Arora M.K. (1997). An evaluation of some factors affecting the accuracy of classification by an artificial neural network. Int. J. Remote Sens..

[21] (2021). Keras. https://keras.io/.

[22] MATLAB (2022). MathWorks. https://www.mathworks.com/products/matlab.html.

[23] Chang C.H., Kao H.Y., Huang S.H. Hardware Implementation for Multiple Activation Functions. Proceedings of the 2019 IEEE International Conference on Consumer Electronics—Taiwan (ICCE-TW).

[24] Moroz L., Samotyy V. (2018). The CORDIC method of calculating the exponential function Metoda CORDIC obliczania funkcji eksponencjalnej. Tech. Trans..

[25] Chang C. (2021). RISC-V P Extension Proposal. https://raw.githubusercontent.com/riscv/riscv-p-spec/master/P-ext-proposal.pdf.

[26] Garofalo A., Rusci M., Conti F., Rossi D., Benini L. (2020). PULP-NN: Accelerating quantized neural networks on parallel ultra-low-power RISC-V processors. Philos. Trans. R. Soc. A.

[27] Dao N., Attwood A., Healy B., Koch D. Flexbex: A risc-v with a reconfigurable instruction extension. Proceedings of the 2020 International Conference on Field-Programmable Technology (ICFPT).

